# Anti-SU Antibody Responses in Client-Owned Cats Following Vaccination against Feline Leukaemia Virus with Two Inactivated Whole-Virus Vaccines (Fel-O-Vax^®^ Lv-K and Fel-O-Vax^®^ 5)

**DOI:** 10.3390/v13020240

**Published:** 2021-02-03

**Authors:** Mark Westman, Jacqueline Norris, Richard Malik, Regina Hofmann-Lehmann, Yasmin A. Parr, Emma Armstrong, Mike McDonald, Evelyn Hall, Paul Sheehy, Margaret J. Hosie

**Affiliations:** 1 Sydney School of Veterinary Science, The University of Sydney, Camperdown, NSW 2006, Australia; jacqui.norris@sydney.edu.au (J.N.); evelyn.hall@sydney.edu.au (E.H.); paul.sheehy@sydney.edu.au (P.S.); 2 Centre for Veterinary Education, The University of Sydney, Camperdown, NSW 2006, Australia; richard.malik@sydney.edu.au; 3 Clinical Laboratory, Department of Clinical Diagnostics and Services, and Center for Clinical Studies, Vetsuisse Faculty, The University of Zurich, CH-8057 Zürich, Switzerland; rhofmann@vetclinics.uzh.ch; 4 MRC—University of Glasgow Centre for Virus Research, Garscube Campus, Bearsden Road, Glasgow G61 1QH, UK; yasmin.parr@glasgow.ac.uk (Y.A.P.); 2190070a@student.gla.ac.uk (E.A.); margaret.hosie@glasgow.ac.uk (M.J.H.); 5 Veterinary Diagnostic Services, The University of Glasgow, Glasgow G61 1QH, UK; mike.mcdonald@glasgow.ac.uk

**Keywords:** Australia, FeLV vaccination, humoral immunity, FeLV infection, vaccine efficacy, veterinary science

## Abstract

A field study undertaken in Australia compared the antibody responses induced in client-owned cats that had been vaccinated using two inactivated whole feline leukaemia virus (FeLV) vaccines, the monovalent vaccine Fel-O-Vax^®^ Lv-K and the polyvalent vaccine Fel-O-Vax^®^ 5. Serum samples from 428 FeLV-uninfected cats (118 FeLV-vaccinated and 310 FeLV-unvaccinated) were tested for anti-FeLV neutralising antibodies (NAb) using a live virus neutralisation assay to identify 378 FeLV-unexposed (NAb-negative) and 50 FeLV-exposed (NAb-positive; abortive infections) cats, following by anti-surface unit (SU) FeLV-A and FeLV-B antibody ELISA testing. An additional 42 FeLV-infected cats (28 presumptively regressively infected, 14 presumptively progressively infected) were also tested for anti-SU antibodies. NAb-positive cats displayed significantly higher anti-SU antibody ELISA responses compared to NAb-negative cats (*p* < 0.001). FeLV-unexposed cats (NAb-negative) that had been vaccinated less than 18 months after a previous FeLV vaccination using the monovalent vaccine (Fel-O-Vax^®^ Lv-K) displayed higher anti-SU antibody ELISA responses than a comparable group vaccinated with the polyvalent vaccine (Fel-O-Vax^®^ 5) (*p* < 0.001 for both anti-FeLV-A and FeLV-B SU antibody responses). This difference in anti-SU antibody responses between cats vaccinated with the monovalent or polyvalent vaccine, however, was not observed in cats that had been naturally exposed to FeLV (NAb-positive) (*p* = 0.33). It was postulated that vaccination with Fel-O-Vax^®^ 5 primed the humoral response prior to FeLV exposure, such that antibody production increased when the animal was challenged, while vaccination with Fel-O-Vax^®^ Lv-K induced an immediate preparatory antibody response that did not quantitatively increase after FeLV exposure. These results raise questions about the comparable vaccine efficacy of the different FeLV vaccine formulations and correlates of protection.

## 1. Introduction

Feline leukaemia virus (FeLV), a member of the *Retroviridae* family, was first reported in 1964 following its discovery by Bill Jarrett and colleagues during the investigation of a time–space cluster of cats with T-cell lymphoma [1]. Subsequent research reported persistently viraemic cats with progressive FeLV infections to be 62 times more likely to develop lymphoma or leukaemia than FeLV-uninfected cats [2,3]. A potential link between transiently viraemic cats with regressive FeLV infections and lymphoma has also been suggested [4,5,6].

In addition to providing an early example of viral oncogenesis, abortive FeLV infections provide one of the few examples of a retroviral infection from which some animals can completely recover. In contrast, other retroviruses such as bovine leukaemia virus, feline immunodeficiency virus (FIV), feline foamy virus, equine infectious anaemia virus, caprine arthritis encephalitis virus and human immunodeficiency virus-1 (HIV-1), cause life-long infections [7]. Additionally, FeLV is one of the few retroviruses for which commercial vaccines exist (in addition to FIV and Jembrana disease in cattle) [8,9,10,11,12,13,14,15,16]. Consequently, the analysis of immune responses facilitating complete recovery in FeLV-exposed cats with abortive infections, and those protecting cats following FeLV vaccination, are important to assist with current efforts to develop effective vaccines against other retroviruses, such as HIV-1 [17,18].

Antibody-mediated (humoral) and cell-mediated immunity are both important in protecting against FeLV infection [8,19]. Following exposure to FeLV, virus-neutralising antibodies (NAbs) directed predominantly against the surface unit (SU) envelope glycoprotein gp70 have been observed in cats with abortive infections, in which FeLV replication is restricted to oropharyngeal tissues before being cleared [20,21,22]. Anti-FeLV NAbs have also been detected in cats with transient viraemia (regressive infections) [19,23,24,25]. Kittens fed colostrum from queens that had recovered from natural FeLV infection, and then subsequently challenged with FeLV, were protected from persistent viraemia (progressive infection) due to the passive transfer of anti-FeLV NAbs [26]. Cats that become persistently viraemic following FeLV challenge typically develop neither NAb nor high levels of FeLV-specific cytotoxic lymphocytes, indicative of inadequate humoral and cell-mediated immune responses, respectively [23,24,25,27]. FeLV vaccination likely primes the humoral and cellular immune responses; thus, anti-FeLV NAbs are usually not detectable in FeLV-vaccinated cats pre-challenge but develop in response to experimental or natural exposure to FeLV [8,9,11,19,21,27,28,29].

The aim of this study was to investigate the anti-SU antibody responses of client-owned cats in Australia that had been vaccinated against FeLV using two commercially available, inactivated whole-virus (IWV) FeLV vaccines (Fel-O-Vax^®^ Lv-K and Fel-O-Vax^®^ 5).

## 2. Materials and Methods

### 2.1. Study Population

Residual samples from a study investigating FeLV infection in healthy client-owned cats, which included client-owned cats from 13 veterinary hospitals around Australia [14], and cats from two rescue facilities in Sydney, New South Wales (NSW) that were sampled in response to FeLV-associated disease outbreaks [21], were utilised.

Animal ethics approval for the sampling of the client-owned cats was granted by the University of Sydney Animal Ethics Committee (Approval number N00/1-2013/3/5920), while the rescue cats were sampled and tested at the request of the facility managers following the diagnosis of progressive FeLV infections.

### 2.2. Vaccination History

Vaccination histories were extracted from the medical records of client-owned cats. FeLV-vaccinated cats had been vaccinated with Fel-O-Vax^®^ Lv-K or Fel-O-Vax^®^ 5 (both vaccines manufactured by Boehringer Ingelheim, Fort Dodge, IA, USA). Five of the 13 veterinary hospitals from which cats were recruited routinely vaccinated at-risk cats against FeLV: two hospitals used monovalent Fel-O-Vax^®^ Lv-K and three used polyvalent Fel-O-Vax^®^ 5. Pre-vaccination FeLV testing was not performed by any of the hospitals. No rescue cats had been vaccinated against FeLV.

Fel-O-Vax^®^ Lv-K is an adjuvanted monovalent FeLV vaccine. This vaccine was administered at the same time as Fel-O-Vax^®^ 3, an adjuvanted polyvalent core vaccine containing feline parvovirus virus (FPV), feline herpesvirus type-1 (FHV-1) and feline calicivirus (FCV), but at a different anatomical site. Fel-O-Vax^®^ 5 (also sold as Fel-O-Vax^®^ Lv-K IV in North America by Elanco Animal Health, Greenfield, IN, USA) is an adjuvanted pentavalent vaccine that contains FeLV, FPV, FHV-1, FCV and *Chlamydia felis*. The FeLV component of both Fel-O-Vax^®^ Lv-K and Fel-O-Vax^®^ 5 is produced as an IWV vaccine from a single subtype isolate (FeLV-A/61E), by the same manufacturer, in the same facility. The antigen potency specifications (minimum and maximum release titres) for FeLV are the same for both Fel-O-Vax^®^ Lv-K and Fel-O-Vax^®^ 5 (Adam Heeley, Technical Services Veterinarian, Boehringer Ingelheim Animal Health Australia *per comm*).

When multiple vaccines were administered concurrently, they were given as separate injections, in different syringes, at different sites. All vaccines were injected subcutaneously into the dorsal interscapular area, except for Fel-O-Vax^®^ Lv-K, which was injected into the right flank fold. In this study, “on-time” vaccination was defined as a primary course of two vaccines given one month apart followed by annual re-vaccination as per manufacturer’s guidelines, with the last FeLV vaccine being administered within 18 months of sampling. Although Fel-O-Vax^®^ Lv-K and Fel-O-Vax^®^ 5 are registered as annual vaccines in Australia, the duration of immunity for FeLV vaccines has been shown to exceed 12 months [10,30,31,32,33,34]. The term “overdue” vaccination was used to define cats for which the last FeLV vaccine had been given more than 18 months prior to sampling.

### 2.3. Determination of FeLV Exposure/Infection Status

A combination of FeLV p27 capsid antigen testing and FeLV proviral real-time polymerase chain reaction (qPCR) testing was used to classify cats as FeLV-uninfected or FeLV-infected, using accepted definitions [35]. FeLV p27 testing of whole blood was performed using three commercially available FeLV point-of-care (PoC) antigen test kits (SNAP Combo^®^, IDEXX Laboratories, Westbrook, ME, USA; Witness^®^, Zoetis Animal Health, Lyon, France; and Anigen Rapid^®^, BioNote, Gyeonggi-do, Korea). FeLV proviral qPCR testing was performed in-house according to a published protocol, using primers designed to amplify a section of the unique region (U3) of the long terminal repeat (LTR) of the three main subtypes of FeLV [35,36].

**Figure 1:**
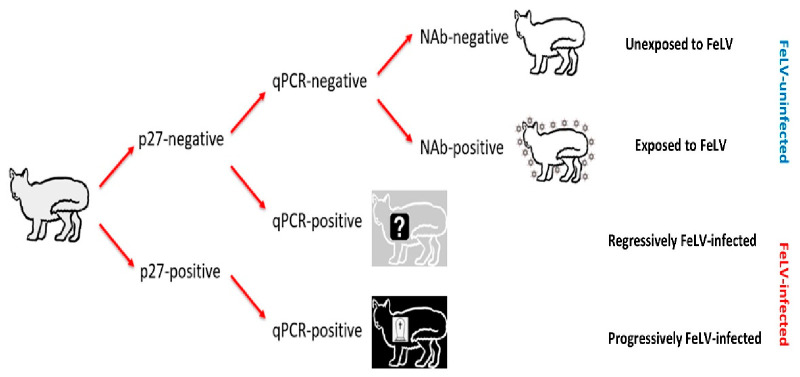
Algorithm used for classifying the FeLV exposure and infection status of cats recruited for the current study (grey cat with black outline). Cats unexposed to FeLV are represented as a white cat with a black outline; FeLV-uninfected cats that had been exposed to FeLV (NAb-positive; abortive infections) are represented as a white cat with a black outline and surrounded by FeLV, to demonstrate their robust immune response to clear early FeLV infection and protect them from further FeLV challenge; presumptively regressively infected cats are represented as a grey cat with a white outline and a question mark to represent a possible predisposition to developing lymphoma; and presumptively progressively infected cats are represented as a black cat with a white outline and a tombstone to represent their poor prognosis. FeLV-infected cats were classified as “presumptively” infected since testing was only performed at a single time point. NAb = neutralising antibody, qPCR = real-time polymerase chain reaction.

Cats testing p27-negative/qPCR-negative were considered FeLV-uninfected, and NAb results were used to further categorise these cats as FeLV-unexposed (NAb-negative) or FeLV-exposed (NAb-positive; abortive infections). FeLV-vaccinated cats testing NAb-positive were classified as abortive infections, since FeLV-vaccinated cats do not produce NAbs prior to exposure to FeLV [8,9,11,19,21,27,28,29]. Cats testing p27-negative/qPCR-positive were considered presumptively regressively infected, while cats testing p27-positive/qPCR-positive were considered presumptively progressively infected (Figure 1). FeLV infection status was considered “presumptive”, since testing had been performed at a single time point and therefore it was not possible to confirm whether viraemia (as determined by p27 positivity) was transient or persistent.

### 2.4. FeLV NAb Testing

NAb were detected using a focus reduction test [29]. Briefly, QN10 cells were incubated overnight in 12-well plates (4 × 10^4^ cells/mL) before the addition of 4 × 10^2^ FFU/mL of FeLV-A/Glasgow-1 that had been incubated for 2 h with an equal volume of two-fold, serial dilutions of plasma samples (1/4, 1/8, 1/16 and 1/32). Residual infectivity was measured in quadruplicate wells. NAb titres were recorded as the reciprocals of the plasma dilutions that visibly reduced the number of focus forming units by 75% compared to the virus control wells incubated without plasma. A NAb titre of 4 was considered weakly NAb-positive, while 32 or greater was considered a strong NAb-positive value.

### 2.5. FeLV Anti-SU Antibody ELISA Testing

Anti-SU antibody ELISA testing was performed to assess humoral response to FeLV vaccination, since FeLV-vaccinated cats have been shown to produce pre-challenge antibodies against whole FeLV and p45 (the non-glycosylated form of gp70) quantifiable by ELISA [37], whereas FeLV-vaccinated cats do not produce NAb without FeLV exposure [8,9,11,19,21,27,28,29]. Plasma samples were tested for anti-SU antibodies, using both Fc-tagged FeLV-A SU and Fc-tagged FeLV-B SU as capture antigens, to ensure a comprehensive analysis of antibody responses against FeLV SU and to maximise test sensitivity [38]. The FeLV-B SU antibody response was interpreted as an antibody response to an epitope shared between FeLV-A and FeLV-B SU.

Positive and negative controls were included on each test plate. The positive control was a pooled sample of plasma from specific pathogen-free (SPF) cats that had recovered from experimental FeLV infection and tested strongly positive for anti-FeLV NAbs. The negative control was a pooled sample of plasma from SPF cats that had negligible reactivity to FeLV SU by immunoblot. Normalised optical density (NOD) values were determined using the formula NOD = [(Sample OD—Negative control OD)/(Positive control OD—Negative control OD)]. Samples were tested in triplicate and tests were repeated if the standard deviation was >0.1. Anti-SU ELISA results were not categorised as “positive” or “negative”; rather, the range of antibody responses against SU were compared among the cats tested.

### 2.6. Determination of FIV Status

Cats were considered FIV-infected if they tested positive for FIV antibodies using two commercially available FIV PoC antibody test kits (Witness^®^, Zoetis Animal Health, Lyon, France; and Anigen Rapid^®^, BioNote, Gyeonggi-do, Korea), which can be used to differentiate FIV-infected and FIV-vaccinated cats [38]. In addition, client-owned cats were tested for FIV proviral DNA using a commercially available qPCR assay (FIV RealPCR^TM^, IDEXX Laboratories, East Brisbane, QLD, Australia), and virus isolation (VI) was performed in rare, discrepant cases (Yamamoto Laboratory, The University of Florida, Gainesville, FL, USA; and Veterinary Diagnostic Services, The University of Glasgow, Scotland, UK) [39,40].

### 2.7. Statistical Analysis

Numerical analyses were performed using the statistical software Genstat 18th Edition (VSN International, Hemel Hempstead, UK) and R Version 3.6.2 (The R Foundation, Vienna, Austria). Ordinal logistic regression (OLR) testing was used to compare sex and breed compositions between groups. A Shapiro–Wilk test was used to assess data for normality. Normally distributed data (cat ages) were analysed using means and two-sample *t*-testing, while non-normal data (anti-SU NOD values) were analysed using medians, Mann–Whitney *U*-testing (paired samples) and restricted maximum likelihood (REML) modelling (multiple groups). Fisher’s exact testing was used to compare binomial outcomes. Statistical significance was considered when *p* < 0.05.

## 3. Results

### 3.1. Sample Population (n = 470)

Residual samples from 470 cats were available for testing (403 client-owned cats, 67 rescue cats) [21]. The signalment details for the entire feline cohort have been described [21]. For the current study, the age, sex and breed of the FeLV-vaccinated/FeLV-uninfected (*n* = 118) and FeLV-unvaccinated/FeLV-uninfected (*n* = 310) groups were similar (*p* = 0.36, 0.95 and 0.25, respectively; 2-sample *t*-testing for age, OLR testing for sex and breed). The age, sex and breed of Fel-O-Vax^®^ Lv-K vaccinated on-time/FeLV-unexposed cats (*n* = 10) and Fel-O-Vax^®^ 5 vaccinated on-time/FeLV-unexposed cats (*n* = 40) were similar (*p* = 0.94, 0.51 and 1.00, respectively), as were the age, sex and breed of Fel-O-Vax^®^ Lv-K vaccinated on-time/FeLV-exposed cats (*n* = 13) and Fel-O-Vax^®^ 5 vaccinated on-time/FeLV-exposed cats (*n* = 26) (*p* = 0.56, 0.51 and 1.00, respectively; 2-sample *t*-testing for age, OLR testing for sex and breed for both comparisons). FeLV-unexposed cats vaccinated on-time with Fel-O-Vax^®^ Lv-K had been re-vaccinated annually 1–7 times, and FeLV-unexposed cats vaccinated on-time with Fel-O-Vax^®^ 5 had been re-vaccinated annually 0–10 times; the median number of annual FeLV vaccinations of both groups was four.

The categorisation of 428 FeLV-uninfected cats and 42 FeLV-infected cats according to FeLV vaccination history and FeLV infection status is shown in Table 1. Four FeLV-infected cats (three presumptively regressive, one presumptively progressive) had been vaccinated against FeLV, although their FeLV infection status prior to primary vaccination had not been determined.

In total, 33 cats were FIV-infected, including four cats co-infected with FIV and FeLV (2 presumptively progressive FeLV infections with FeLV cycle threshold (C_T_) values of 16.8 and 21.3, respectively; 2 presumptively regressive FeLV infections with C_T_ values of 31.2 and 37.2, respectively).

### 3.2. FeLV NAb Testing

Seventy-eight samples tested NAb-positive, including 50 FeLV-uninfected cats (abortive infections; 39 cats FeLV-vaccinated on-time, four overdue for FeLV vaccination and seven FeLV-unvaccinated cats), 26 presumptively regressive infections and two presumptively progressive infections (that likely represented one early regressive infection that was still antigenaemic and one focal infection [21]). The remaining 392 samples tested NAb-negative. Presumptively regressive infections tended to test NAb-positive whereas presumptively progressive infections tended to test NAb-negative (26/28 (93%) vs. 2/14 (14%); *p* < 0.0001; Fisher’s exact test) (Table 1). 

A higher proportion of FeLV-vaccinated/FeLV-uninfected cats tested NAb-positive compared to FeLV-unvaccinated/FeLV-uninfected cats (43/118 (36%) vs. 7/310 (2%); *p* < 0.0001; Fisher’s exact test). There was no difference in exposure rate between the monovalent vaccine group (Fel-O-Vax^®^ Lv-K) and the polyvalent vaccine group (Fel-O-Vax^®^ 5), irrespective of whether cats overdue for vaccination or vaccinated/infected cats were included, suggesting a similar risk of FeLV exposure for both groups (10/23 (43%) vs. 40/66 (61%) for on-time vaccination, 14/28 (50%) vs. 61/90 (68%) including on-time/overdue vaccination, and 14/29 (48%) vs. 61/93 (66%) including vaccinated/infected cats; *p* = 0.22, 0.12 and 0.13, respectively; Fisher’s exact tests). Both monovalent and polyvalent vaccinated/exposed cats (abortive infections) displayed a range of NAb titres (Table 2). There was no significant difference between the monovalent and polyvalent vaccines in vaccinated/exposed cats according to reciprocal NAb titre (4—*p* = 0.59; 8—*p* = 0.94; 16—*p* = 0.96; ≥32—*p* = 0.37; 2-sample binomial tests).

### 3.3. FeLV Anti-SU Antibody ELISA Testing by NAb Result

**Figure 2:**
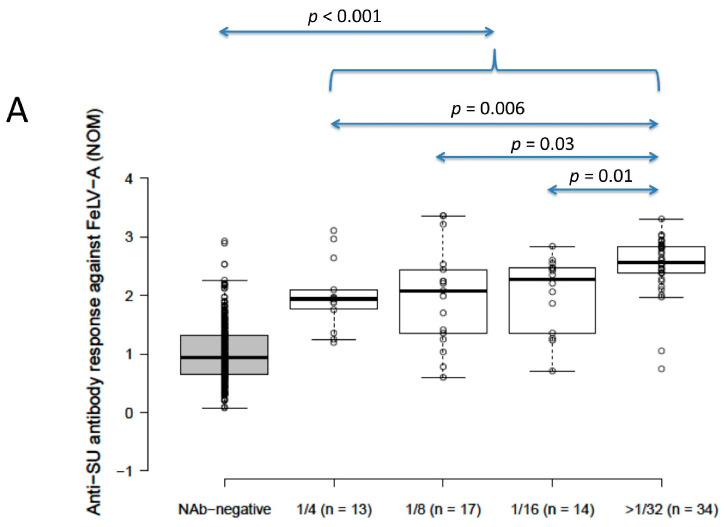

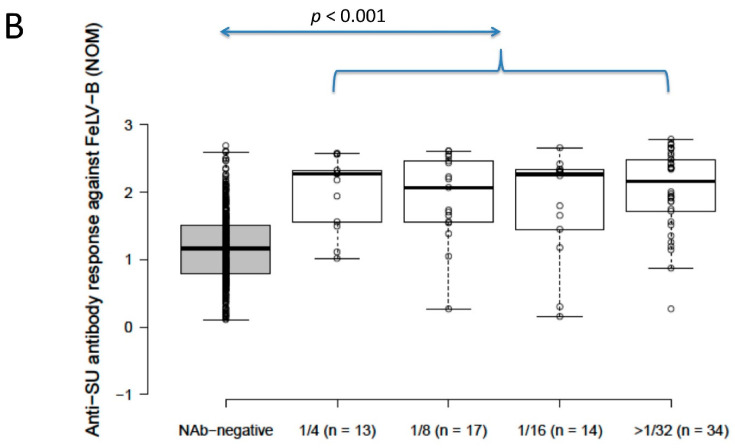
Results from anti-SU antibody ELISA testing according to NAb titre ((**A**) anti-FeLV-A SU antibody ELISA results, (**B**) anti-FeLV-B SU antibody ELISA results). Anti-SU antibody levels against FeLV-A for strongly neutralising samples (≥32) were significantly higher than other NAb-positive samples (i.e., 4, 8 and 16) (*p* = 0.03; REML testing), but anti-SU antibody levels against FeLV-B were not significantly different between NAb-positive groups (*p* = 0.32; REML testing). All NAb-positive groups had significantly higher median anti-SU antibody levels against both FeLV-A and FeLV-B SU than NAb-negative samples (*p* < 0.001; least significant difference testing). Data points are plotted as open circles, centre lines show the medians, box limits indicate the 25th and 75th percentiles, and whiskers extend 1.5 times the interquartile range from the 25th and 75th percentiles. NOD = normalised optical density, NAb = neutralising antibody, SU = surface unit.

Anti-SU antibody ELISA levels for NAb-positive cats (*n* = 78) were significantly higher than for NAb-negative cats (*n* = 392) (2.4 vs. 0.9 for FeLV-A, 2.2 vs. 1.2 for FeLV-B; *p* < 0.001 for both; Mann–Whitney *U*-tests). Anti-SU antibody levels against FeLV-A for strongly neutralising samples (≥32) were significantly higher than other NAb-positive samples grouped according to reciprocal titre (i.e., 4, 8 and 16; NOD values were 1.9, 2.1, 2.3 and 2.6 for 4, 8, 16 and ≥32, respectively) (*p* = 0.03; REML testing). Anti-SU antibody ELISA levels against FeLV-B were not significantly different between NAb-positive samples grouped according to reciprocal titre (NOD values were 2.3, 2.1, 2.3 and 2.2 for 4, 8, 16 and ≥32, respectively) (*p* = 0.32; REML testing). All NAb-positive groups, when considered individually (i.e., 4, 8, 16 and 32), were significantly higher against both FeLV-A and FeLV-B SU than NAb-negative samples (*p* < 0.001; least significant difference testing) (Figure 2).

The positive control sample included on each plate had a mean absorbance of 2.6 and the negative control had a mean OD of 0.5.

### 3.4. FeLV Anti-SU Antibody ELISA Testing in Unvaccinated Cats and Abortive Infections

**Figure 3:**
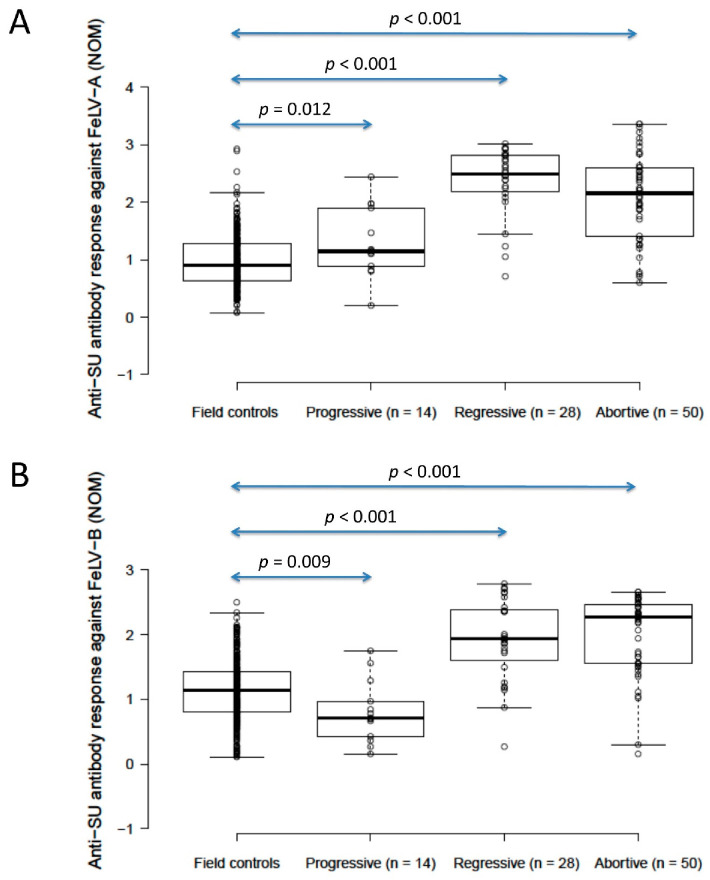
Comparison of anti-SU antibody ELISA responses to examine the effect of FeLV exposure/infection ((**A**) anti-FeLV-A SU antibody ELISA results, (**B**) anti-FeLV-B SU antibody ELISA results). Anti-SU ELISA results likely reflect differences between groups with regard to virus-neutralising antibody (NAb) titres. The field control group consisted of unvaccinated and unexposed cats (*n* = 303). The FeLV-infected cats consisted of presumptively progressive infections (*n* = 14) and presumptively regressive infections (*n* = 28). The abortive group (uninfected/exposed; *n* = 50) consisted of cats vaccinated with Fel-O-Vax^®^ 5 (*n* = 29), cats vaccinated with Fel-O-Vax^®^ Lv-K (*n* = 14) and FeLV-unvaccinated cats (*n* = 7). Statistically significant differences in median NOD values are shown (Mann–Whitney *U*-tests). Data points are plotted as open circles, centre lines show the medians, box limits indicate the 25th and 75th percentiles, and whiskers extend 1.5 times the interquartile range from the 25th and 75th percentiles. Results from vaccinated cats are shown in Figure 4. NOD = normalised optical density, SU = surface unit.

The anti-SU antibody ELISA levels against FeLV-A of unvaccinated/unexposed cats (*n* = 303; considered to be the field control group) were significantly lower than those of presumptively progressively infected cats (*n* = 14) (0.9 vs. 1.1, *p* = 0.012; Mann–Whitney *U*-test), but unvaccinated/unexposed cats had significantly higher anti-SU antibody ELISA levels against FeLV-B than presumptively progressively infected cats (1.1 vs. 0.7, *p* = 0.009; Mann–Whitney *U*-test). A wide range of anti-SU ELISA values was observed in this field control group (0.07–2.9 against FeLV-A, 0.1–2.5 against FeLV-B). Presumptively regressive infections (*n* = 28) had significantly higher anti-SU antibody ELISA levels (2.5/1.9 against FeLV-A/FeLV-B) than both unvaccinated/unexposed cats and presumptively progressively infected cats (*p* < 0.001 for both; Mann–Whitney *U*-tests). Anti-SU antibody ELISA responses were similar between presumptively regressive infections and pooled vaccinated/unvaccinated abortive infections (*n* = 50; 2.2/2.3 against FeLV-A/FeLV-B) (*p* = 0.10 for FeLV-A, *p* = 0.62 for FeLV-B; Mann–Whitney *U*-tests; Figure 3). Excluding anti-SU antibody results from four infected cats (three regressive, one progressive) that were vaccinated, to exclude possible confounding effects of vaccination and infection on antibody production, had no effect on the findings.

### 3.5. FeLV Anti-SU Antibody ELISA Testing in Vaccinated Cats

**Figure 4:**
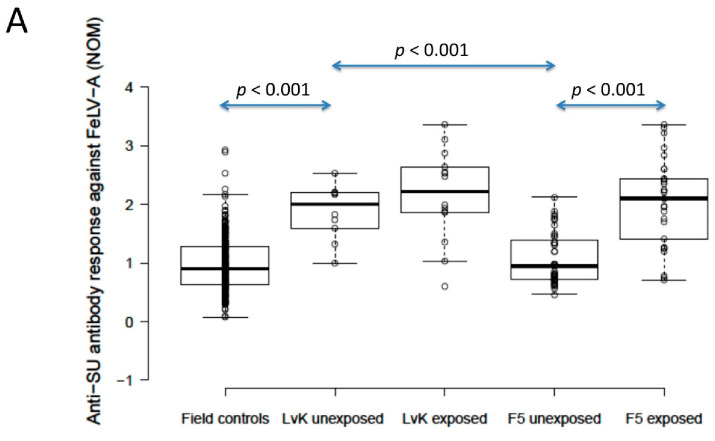

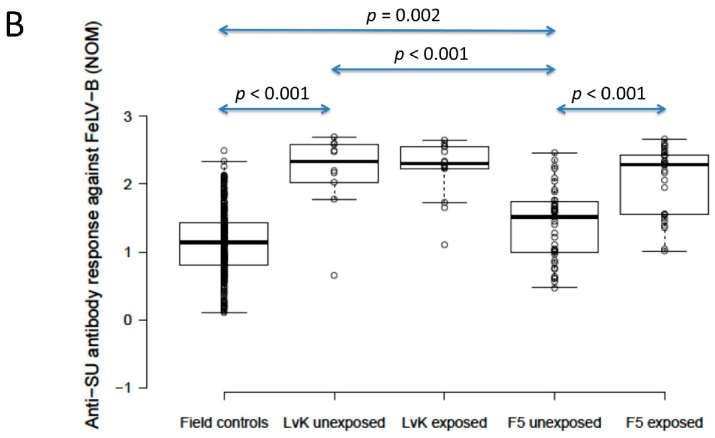
Comparison of anti-SU antibody ELISA responses to examine the effect of FeLV vaccination and natural exposure to FeLV following vaccination ((**A**) anti-FeLV-A SU antibody ELISA results, (**B**) anti-FeLV-B SU antibody ELISA results). The field control group consisted of unvaccinated and unexposed cats (*n* = 303) and is included to provide a baseline. Ten unexposed cats had been vaccinated on-time with Fel-O-Vax^®^ Lv-K and 40 unexposed cats had been vaccinated on-time with Fel-O-Vax^®^ 5. In total, 43 vaccinated/uninfected cats had been exposed to FeLV (abortive infections), including 14 cats vaccinated with Fel-O-Vax^®^ Lv-K and 29 cats vaccinated with Fel-O-Vax^®^ 5. Statistically significant differences in median NOD values between vaccinated groups after exposure are shown. The monovalent vaccine (Fel-O-Vax^®^ Lv-K) induced significantly higher antibody levels in unexposed cats against both FeLV-A and FeLV-B SU than the polyvalent vaccine (Fel-O-Vax^®^ 5) (*p* < 0.001 for both; Mann–Whitney *U*-tests). Cats vaccinated with the polyvalent Fel-O-Vax^®^ 5 vaccine and exposed to FeLV displayed a booster effect of natural FeLV exposure (*p* < 0.001 for both FeLV-A and FeLV-B SU; Mann–Whitney *U*-tests); cats vaccinated with the monovalent Fel-O-Vax^®^ Lv-K vaccine and exposed to FeLV displayed no such booster effect (*p* = 0.33 for FeLV-A and *p* = 0.82 for FeLV-B; Mann–Whitney *U*-tests). Data points are plotted as open circles, centre lines show the medians, box limits indicate the 25th and 75th percentiles, and whiskers extend 1.5 times the interquartile range from the 25th and 75th percentiles. NOD = normalised optical density, SU = surface unit, LvK = Fel-O-Vax^®^ Lv-K, F5 = Fel-O-Vax^®^ 5.

The anti-SU antibody ELISA levels against FeLV-A of polyvalent FeLV-vaccinated (Fel-O-Vax^®^ 5)/unexposed cats (*n* = 40) were not significantly different from unvaccinated/unexposed cats (*n* = 303) (0.9 vs. 0.9; *p* = 0.18; Mann–Whitney *U*-test), but FeLV-vaccinated (Fel-O-Vax^®^ 5)/unexposed cats had significantly higher anti-SU antibody responses against FeLV-B compared to unvaccinated/unexposed cats (1.5 vs. 1.1); *p* = 0.002; Mann–Whitney *U*-test). Anti-SU antibody ELISA levels of monovalent FeLV-vaccinated (Fel-O-Vax^®^ Lv-K)/unexposed cats (*n* = 10) were significantly higher than unvaccinated/unexposed cats (*n* = 303) for both FeLV-A and FeLV-B (2.0 vs. 0.9 and 2.3 vs. 1.1, respectively; *p* < 0.001 for both; Mann–Whitney *U*-tests) (Figure 4).

### 3.6. Comparing FeLV Anti-SU Antibody Responses between Fel-O-Vax^®^ Lv-K and Fel-O-Vax^®^ 5

When unexposed cats vaccinated on-time with the two FeLV vaccines were compared, the monovalent vaccine (Fel-O-Vax^®^ Lv-K) induced significantly higher antibody levels against both FeLV-A and FeLV-B SU than the polyvalent vaccine (Fel-O-Vax^®^ 5) (2.0 vs. 0.9 and 2.3 vs. 1.5; *p* < 0.001 for both; Mann–Whitney *U*-tests) (Figure 4). These findings were unchanged if the definition of on-time vaccination was changed from 18 months to 12 months since last FeLV vaccination.

Cats vaccinated with the polyvalent Fel-O-Vax^®^ 5 vaccine and exposed to FeLV (*n* = 29, including three cats overdue for vaccination) had significantly higher anti-SU antibody ELISA levels against both FeLV-A and FeLV-B compared to cats vaccinated on-time with Fel-O-Vax^®^ 5 and unexposed (*n* = 40), consistent with a booster effect of natural FeLV exposure (2.1 vs. 0.9 for FeLV-A and 2.3 vs. 1.5 for FeLV-B; *p* < 0.001 for both; Mann–Whitney *U*-tests). No booster effect was observed in cats vaccinated with the monovalent Fel-O-Vax^®^ Lv-K and exposed to FeLV (*n* = 14, including one cat overdue for vaccination) compared to cats vaccinated on-time with Fel-O-Vax^®^ Lv-K and unexposed (*n* = 10) (2.2 vs. 2.0 for FeLV-A and 2.3 vs. 2.3 for FeLV-B; *p* = 0.33 and 0.82; Mann–Whitney *U*-tests) (Figure 4).

When vaccinated/exposed cats were compared, there was no significant difference in antibody response against either FeLV-A or FeLV-B SU between cats vaccinated with the two different FeLV vaccines (*p* = 0.65 and 0.33, respectively; Mann–Whitney *U*-tests).

Unfortunately, group sizes were too small to compare vaccinated/unexposed and vaccinated/exposed cats based on the number of annual FeLV re-vaccinations administered. 

## 4. Discussion

In this study, anti-FeLV SU antibody responses were measured by ELISA, comparing the results between FeLV-vaccinated and FeLV-unvaccinated cats given core vaccines concurrently under natural conditions. Surprisingly, despite the two IWV FeLV vaccines being derived from the same cell line and strain of FeLV (FeLV-A/61E), using the same adjuvant formulation and being produced by a single manufacturer in the same facility [41], a quantitatively different humoral response was observed. 

Vaccination using the monovalent FeLV vaccine (Fel-O-Vax^®^ Lv-K, given concurrently with Fel-O-Vax^®^ 3) induced significantly higher anti-SU antibody levels in FeLV-unexposed cats against both FeLV-A and FeLV-B compared to vaccination using a polyvalent vaccine that included a FeLV component (Fel-O-Vax^®^ 5). Following natural exposure to FeLV, however, antibody responses to the different vaccines were similar. While vaccination with the polyvalent Fel-O-Vax^®^ 5 reproduced the response reported from experimental studies, in which FeLV vaccination led to minimal antibody production but primed the cat’s humoral immune system in case of FeLV exposure [19], vaccination with the monovalent Fel-O-Vax^®^ Lv-K resulted in a robust humoral response irrespective of FeLV exposure. Similarly, a strong humoral immune response following vaccination (and prior to experimental FeLV challenge) with a recombinant p45 FeLV vaccine (Leucogen^®^) has been observed, using an ELISA to measure anti-p45 antibodies [42].

This is the first time that a difference in antibody response between cats vaccinated with Fel-O-Vax^®^ Lv-K and Fel-O-Vax^®^ 5 has been reported. Previously, most Fel-O-Vax^®^ vaccine studies have only considered one of the two vaccine formulations at a time and have been primarily concerned with vaccine efficacy as determined by the presence or absence of antigenaemia or viraemia after challenge (Table 3). Only two groups to date have concurrently reported the efficacies of the monovalent and polyvalent Fel-O-Vax^®^ FeLV vaccines, but neither measured anti-FeLV antibody levels following vaccination or FeLV challenge [30,41,43]. The effect of FIV infection on the anti-SU antibody response following FeLV vaccination in the current study could not be examined because of insufficient sample sizes, although it was reported that healthy experimentally FIV-infected cats were able to mount a sufficient humoral immune response to vaccination with a recombinant FeLV vaccine in the early phase of FIV infection [42]. 

Any anti-FeLV SU antibody result should be considered alongside results from p27 antigen and proviral PCR testing, particularly when testing has only been performed at a single time point [44]. As predicted, presumptively progressively infected cats in the current study had low anti-SU antibody levels, indicative of poor humoral (and presumably cell-mediated) immune responses despite ongoing viraemia (antigenaemia). There was a wide range of anti-SU responses detected by ELISA for the field control group (unvaccinated and unexposed); we suspect that this spread of results could have reflected anti-SU antibodies that were detected by ELISA but did not neutralise the virus used in the NAb assay, since it was demonstrated previously that some FeLV-infected cats produce antibodies that do not contribute to virus neutralisation [45]. It is likely that the field control group contained some cats that had recovered from FeLV exposure after developing protective cellular immunity mediated by FeLV-specific cytotoxic T lymphocytes, in the absence of NAb, while developing antibodies that were detectable by ELISA [19,23,24]. Presumptively regressively infected cats displayed higher anti-SU antibody levels than presumptively progressively infected cats and the field control group, and similar levels to the antibody responses in uninfected/exposed cats (abortive infections), providing evidence of strong and effective humoral immune responses in naturally infected cats with regressive FeLV infections [21,22,28,44,46]. This conclusion was also supported by the higher proportion of presumptively regressively infected cats that tested positive for anti-FeLV NAbs, compared to only a small number of presumptively progressively infected cats.

The reason for the difference in antibody response observed between cats vaccinated with the monovalent FeLV and polyvalent FeLV vaccine is not known. A saturation phenomenon with the anti-SU antibody ELISA, that might have limited higher NOD values (e.g., for cats vaccinated with Fel-O-Vax^®^ Lv-K and then naturally exposed to FeLV), was considered unlikely on the basis of internal testing that demonstrated a concentration dependent decrease in absorbance levels, even with a range of starting sample dilutions. This finding contrasts with a study that compared the immunogenicity of a recombinant FeLV p45 vaccine given as a monovalent or polyvalent formulation (Nobivac^®^ FeLV vs. lyophilised Nobivac^®^ Forcat reconstituted in one dose of Nobivac^®^ FeLV), reporting that both vaccinations induced comparable antibody levels against FeLV as measured by an anti-p45 antibody ELISA [47]. One possible explanation for the unexpected results from the current study is that the route of vaccine administration impacted the antibody response, since Fel-O-Vax^®^ Lv-K was injected into the right flank fold and Fel-O-Vax^®^ 5 was injected into the dorsal interscapular region. In humans, a reduced seroconversion rate (up to 17-fold) was reported when an intramuscular hepatitis B vaccine was administered in the buttock rather than the arm, with delayed vaccine antigen release, or a lower number of macrophages, T and B lymphocytes in the injected area, hypothesised to be responsible for the difference in antibody response [48]. In rats, the highest antibody levels following vaccination with a commercial core canine vaccine were found in animals vaccinated subcutaneously in the *houhai* acupuncture site (the dorsal midline between the anus and tail base), and the lowest antibody levels were in animals vaccinated subcutaneously in the back at the level of the last thoracic vertebra on the dorsal midline [49]. Additional imaging investigations by the same researchers suggested that the enhanced humoral response observed in rats was as a result of increased lymphocyte activation and drainage in the *houhai acupoint* compared to the dorsal interscapular area [50]. To the best of the authors’ knowledge, no studies have investigated differences in anti-FeLV antibody responses in cats associated with the site of vaccination, in part because vaccine licensing studies usually involve administering vaccinations subcutaneously into the interscapular region [33]. The European Advisory Board on Cat Diseases (ABCD) guidelines on feline injection-site sarcoma recommend that veterinarians should avoid administering any vaccinations subcutaneously into the interscapular region. Vaccination in the distal limb or tail is recommended as surgery is more likely to be curative if a sarcoma develops at one of those sites compared to the interscapular area [51,52].

A second possible explanation for this unexpected finding is that an adjuvant effect was observed, with approximately two times the volume of adjuvant inducing higher anti-FeLV antibody responses (total adjuvant volume when Fel-O-Vax^®^ 3 and Fel-O-Vax^®^ Lv-K given concurrently was 2 mL vs. 1 mL with Fel-O-Vax^®^ 5). A previous investigation of Fel-O-Vax^®^ Lv-K efficacy reported a clear association between adjuvant concentration and vaccine efficacy [41]. Furthermore, in this study, 8/10 FeLV-unexposed cats vaccinated on-time with Fel-O-Vax^®^ Lv-K and Fel-O-Vax^®^ 3 were also concurrently vaccinated against FIV (Fel-O-Vax^®^ FIV; Boehringer Ingelheim, Fort Dodge, IA, USA), making a total adjuvant volume administered of 3 mL. Conversely, no difference was observed between FeLV-unexposed cats concurrently vaccinated with Fel-O-Vax^®^ 5 and Fel-O-Vax^®^ FIV (i.e., total adjuvant volume 2 mL) and cats vaccinated with Fel-O-Vax^®^ 5 only (i.e., adjuvant volume 1 mL). The sample size was too small to compare the antibody responses of Fel-O-Vax^®^ Lv-K vaccinated cats with and without the administration of Fel-O-Vax^®^ FIV to examine the effect of adjuvant volume in these cats. To further explore the possibility of an adjuvant volume effect, it would be useful to measure antibody responses against FPV, FHV-1 and FCV in the different FeLV-vaccinated groups and to determine whether an enhanced humoral immune response was a generalised phenomenon.

A third possible explanation for the difference in antibody responses between the monovalent and polyvalent FeLV vaccine may have been a “batch effect”. Different batches of FeLV vaccines are known to vary within a range of approved potency values that have been demonstrated to be safe and efficacious. It is possible, especially given the low rate of FeLV vaccination in Australia, that the two veterinary hospitals using Fel-O-Vax^®^ Lv-K (located approximately 5 km apart, owned by the same veterinarian, but run separately including stock ordering) used FeLV vaccines from the same highly immunogenic batch during the three-year study period. Unfortunately, vaccine batch numbers were not recorded in the medical records.

The phenomenon of “antigenic competition” was also considered as a potential explanation for the weaker antibody response that was observed in FeLV-unexposed cats vaccinated with the polyvalent FeLV vaccine compared to the monovalent FeLV vaccine. Antigenic competition has been reported in sheep vaccinated against the foot rot pathogen *Dichelobacter nodosus*. With this example, however, interference was associated with immunologically related pilus antigens, and a high level of protection with the polyvalent vaccine against all nine antigens was still achieved [55]. We hypothesise that a competitive effect would not be anticipated in a polyvalent vaccine such as Fel-O-Vax^®^ 5 that contains five antigenically distinct organisms [56]. Furthermore, Fel-O-Vax^®^ Lv-K vaccinated cats also received a killed-adjuvanted trivalent core vaccine (Fel-O-Vax^®^ 3) concurrently, but at a different site. Although antigenic competition is theoretically more likely to occur with Fel-O-Vax^®^ 5 than concurrent Fel-O-Vax^®^ 3/Fel-O-Vax^®^ Lv-K administration, due to the local component of antigenic competition, there is no published evidence that suggests that administering multiple antigens mixed in the same syringe (e.g., Fel-O-Vax^®^ 5) decreases the antibody response in vaccinated cats. It seems conceivable, however, that the pronounced difference in humoral immunogenicity observed in the present study with the administration of two different vaccine formulations, both containing the same FeLV IWV antigen potency, could have been the result of the monovalent vaccine (Fel-O-Vax^®^ Lv-K) being administered to a different area of the cat (right flank fold) and a different draining lymph node to the other vaccine antigens.

Further research is required, testing larger cohorts of cats, to determine if the observed difference in antibody response between cats vaccinated using Fel-O-Vax^®^ Lv-K and Fel-O-Vax^®^ 5 is correlated with a difference in protection from natural FeLV challenge. Since FeLV exposure/infection status had not been ascertained prior to FeLV vaccination in any cats, including for the four FeLV-vaccinated/FeLV-infected cats (three regressive, one progressive), due to the retrospective nature of the current study, inferences could not be made about FeLV vaccine effectiveness in the field. To date, only laboratory-based studies involving SPF cats, rather than field-based vaccine efficacy studies, have been performed to report the preventative fractions of Fel-O-Vax^®^ Lv-K and Fel-O-Vax^®^ 5, involving different challenge viruses and routes of inoculum administration (Table 3) [11,27,28,30,41,43,53,54]. In one of only two studies to directly compare the efficacy of Fel-O-Vax^®^ Lv-K and Fel-O-Vax^®^ Lv-K IV (identical to Fel-O-Vax^®^ 5), Legendre et al. (1991) (with results reported in Sebring et al. 1991) demonstrated that no kittens in either group, vaccinated subcutaneously in the flank region, became progressively infected, and both FeLV vaccines demonstrated preventative fractions of 100% [41,43]. The second study to examine both vaccine formulations reported efficacies of 86% and 100% for Fel-O-Vax^®^ Lv-K and Fel-O-Vax^®^ Lv-K IV, respectively, but did not perform any statistical analysis to assess the possible significance of this difference [30]. 

The efficacies of FeLV vaccines other than the Fel-O-Vax^®^ range, administered as either monovalent or polyvalent formulations, have been shown to be comparable. For example, in a study to determine the efficacy of Versifel^®^ FeLV (Zoetis Animal Health; an IWV vaccine containing FeLV-A, FeLV-B and FeLV-C), administered at the same time as a modified live-virus (MLV) trivalent core vaccine, no difference in FeLV vaccine efficacy was observed when the two vaccines were given concurrently (the FeLV component was injected subcutaneously at the base of the neck and the MLV component was injected subcutaneously in the left thoracic wall) compared to simultaneous administration (the MLV component was reconstituted using the FeLV vaccine and the entire contents were administered subcutaneously at the base of the neck) [57]. Similarly, in a study investigating the efficacy of a canarypox virus-vectored FeLV vaccine (Purevax^®^ FeLV, Merial), no difference in vaccine efficacy was observed, whether the FeLV component was administered as a monovalent or polyvalent vaccine [58]. Field-based vaccine efficacy studies for all FeLV vaccines are required to determine vaccine performance under natural challenge conditions and in different jurisdictions where different FeLV strains are circulating, similar to studies testing the efficacy of Fel-O-Vax^®^ FIV in the field [14,59].

## 5. Conclusions

In many countries around the world, including Australia, the prevalence of FeLV infection has decreased in part due to the use of efficacious vaccines. In other countries, including many in Asia, South America and some parts of Europe, the prevalence of FeLV remains high and vaccination programs are urgently required. It will be important to determine whether the increased humoral antibody response against the FeLV SU observed in FeLV-unexposed cats vaccinated with the monovalent FeLV vaccine (Fel-O-Vax^®^ Lv-K) compared to the polyvalent vaccine (Fel-O-Vax^®^ 5) is correlated with increased efficacy, and to address whether the site of vaccine administration affects the anti-SU antibody response and hence the efficacy of vaccination. Encouragingly, after natural exposure to FeLV, FeLV-vaccinated cats had comparable anti-SU antibody levels, suggesting that vaccination with either the monovalent or polyvalent Fel-O-Vax^®^ FeLV vaccine initiates a strong and protective antibody response on challenge. Veterinarians should continue to vaccinate any cat likely to be exposed to FeLV-infected cats, particularly kittens and young adult cats in multi-cat situations, to reduce the risk of progressive FeLV infection and the development of disease. FeLV testing prior to vaccination is advisable and will assist studies of the efficacy of FeLV vaccination in the field.

## Figures and Tables

**Table 1:** Summary of FeLV infection status and FeLV vaccination history of the cats in the current study (*n* = 470). On-time vaccination referred to the previous vaccination being given within the past 18 months, while overdue vaccination referred to the last vaccination being given more than 18 months previously. FeLV-uninfected/FeLV-unexposed cats that were overdue for vaccination were excluded from all analyses, while FeLV-uninfected/FeLV-exposed cats (abortive infections) overdue for vaccination were retained. None of the four FeLV-vaccinated/FeLV-infected cats had been tested for FeLV infection prior to vaccination.

FeLV Infection Status	Category	FeLV Vaccination Status				
		FeLV-Unvaccinated	On-Time Fel-O-Vax® Lv-K	On-Time Fel-O-Vax® 5	Overdue Fel-O-Vax® Lv-K	Overdue Fel-O-Vax® 5
FeLV-uninfected (*n* = 428)	FeLV-unexposed (*n* = 378) (p27-negative, qPCR-negative, NAb-negative)	303	10	40	4	21
	FeLV-exposed (abortive infections) (*n* = 50) (p27-negative, qPCR-negative, NAb-positive)	7	13	26	1	3
FeLV-infected (*n* = 42)	Presumptively regressive infections (*n* = 28) (p27-negative, qPCR-positive)	25	1 ^1^	1 ^2^	0	1 ^3^
	Presumptively progressive infections (*n* = 14) (p27-positive, qPCR-positive)	13	0	1 ^4^	0	0

^1^ Vaccinated with Fel-O-Vax^®^ Lv-K, three months prior to sampling. ^2^ Vaccinated with Fel-O-Vax^®^ 5, nine months prior to sampling. ^3^ Vaccinated with Fel-O-Vax^®^ 5, 136 months prior to sampling. ^4^ Vaccinated with Fel-O-Vax^®^ 5, two months prior to sampling.

**Table 2:** Summary of NAb results in vaccinated/exposed cats (abortive infections), arranged by vaccination type and reciprocal titre. A NAb titre of 4 was considered weakly NAb-positive while a NAb titre of 32 or greater was considered strongly NAb-positive. Cats overdue for vaccination were included (one vaccinated with Fel-O-Vax^®^ Lv-K, three vaccinated with Fel-O-Vax^®^ 5). NAb = neutralising antibody.

	NAb Result			
	4	8	16	≥32
Monovalent vaccine (Fel-O-Vax^®^ Lv-K; *n* = 14)	5 (36%)	5 (36%)	3 (21%)	1 (7%)
Polyvalent vaccine (Fel-O-Vax^®^ 5; *n* = 29)	8 (28%)	10 (34%)	6 (21%)	5 (17%)

**Table 3:** Summary of vaccine efficacy studies for Fel-O-Vax^®^ Lv-K and Fel-O-Vax^®^ 5 (marketed as Fel-O-Vax^®^ Lv-K IV in the USA). Preventative fraction is defined as ([percentage persistent viraemia in controls]—[percentage persistent viraemia in vaccinates]/percentage persistent viraemia in controls ×100). Since both Fel-O-Vax^®^ Lv-K and Fel-O-Vax^®^ 5 are inactivated whole-virus vaccines derived from the same seed virus (FeLV-A/61E), challenge with FeLV-A/61E virus is considered homologous FeLV challenge, while challenge with a strain other than FeLV-A/61E is considered heterologous FeLV challenge. IP = intraperitoneal, ON = oronasal, IN = intranasal, SC = subcutaneous.

Reference	Vaccine	How Vaccine Given	Challenge Information		Number of Cats Infected		Preventative Fraction
			FeLV Strain	Route	Vaccinates	Controls	
Torres et al. (2010) [11]	Fel-O-Vax^®^ Lv-K	Not stated	FeLV-A/61E	IP (4 months after last vaccination)	0/8	7/8	100%
Grosenbaugh et al. (2006) [53]	Fel-O-Vax^®^ Lv-K	SC, caudolateral thigh	FeLV-A/61E	ON (4 weeks after last vaccination, for 2 consecutive days)	1/11	10/10	91%
Torres et al. (2005) [27]	Fel-O-Vax^®^ Lv-K	SC, location not stated	FeLV-A/61E	ON (3 weeks after last vaccination)	1/10	7/10	86%
Grosenbaugh et al. (2004) [54]	Fel-O-Vax^®^ Lv-K	SC, caudolateral thigh	FeLV-A/61E	ON (4 weeks after last vaccination, for 2 consecutive days)	0/10	9/10	100%
Hoover et al. (1996) [30]	Fel-O-Vax^®^ Lv-K	Not stated	FeLV-A/61E	Not stated	13% ^1^	92% ^1^	86% ^1^
Legendre et al. (1991) [43]	Fel-O-Vax^®^ Lv-K	SC in the flank	At least four different strains of FeLV-A (two laboratory strains and at least two field strains)	In-contact (2 weeks after last vaccination, for 31 weeks)	0/12	7/11	100%
Sebring et al. (1991) [41]	Fel-O-Vax^®^ Lv-K	Not stated	Not stated	IP (2 weeks after last vaccination), then in-contact with challenged controls for 72 days)	4/94	57/62	95% ^2^
Hofmann-Lehmann et al. (2006) [28]	Fel-O-Vax^®^ Lv-K IV (Fel-O-Vax^®^ 5)	SC, location not stated	FeLV subtype A/Glasgow-1	IP (4 weeks after last vaccination)	5/10	9/10	44%
Hoover et al. (1996) [30]	Fel-O-Vax^®^ Lv-K IV (Fel-O-Vax^®^ 5)	SC (not stated where)	FeLV-A/61E	IN (one year after last vaccination)	0/5	10/10	100% ^3^
Sebring et al. (1991) [41]	Fel-O-Vax^®^ Lv-K IV (Fel-O-Vax^®^ 5)	SC in the flank	At least four different strains of FeLV-A (two laboratory strains and at least two field strains)	In-contact (2 weeks after last vaccination, for 31 weeks)	0/11	7/11	100% ^4^

^1^ Exact study numbers were not reported in the manuscript. A total of 150 specific pathogen-free cats were tested. The primary author was contacted and asked for more detail about the site of vaccination, group numbers and challenge route; unfortunately, the data were no longer available. ^2^ Results pooled from four different studies. The first two studies reported in this paper are not included in the table. The first study involved intramuscular vaccination (0/5 vaccinates became FeLV-infected following IP challenge two weeks after the second vaccination, 4/7 control cats became FeLV-infected). The second study involved varying doses of adjuvant in the vaccine to help determine the optimal vaccine constitution. ^3^ Five additional cats were vaccinated intramuscularly and are not included in the table. None of these five cats became FeLV-infected. ^4^ Study performed by Legendre et al. (1991) [43], reported by Sebring et al. (1991) [41].

## References

[1] Jarrett W.F.H., Crawford E.M., Martin W.B., Davie F. (1964). Leukaemia in the cat: A virus-like particle associated with leukaemia (lymphosarcoma). Nature.

[2] Shelton G.H., Grant C.K., Cotter S.M., Gardner M.B., Hardy W.D., DiGiacomo R.F. (1990). Feline immunodeficiency virus and feline leukemia virus infections and their relationships to lymphoid malignancies in cats: A retrospective study (1968–1988). J. Acquir. Immune. Defic. Syndr..

[3] Cristo T.G., Biezus G., Noronha L.F., Pereira L., Withoeft J.A., Furlan L.V., Costa L.S., Traverso S.D., Casagrande R.A. (2019). Feline lymphoma and a high correlation with feline leukaemia virus infection in Brazil. J. Comp. Pathol..

[4] Gabor L.J., Jackson M.L., Trask B., Malik R., Canfield P.J. (2001). Feline leukaemia virus status of Australian cats with lymphosarcoma. Aust. Vet. J..

[5] Jackson M.L., Haines D.M., Meric S.M., Misra V. (1993). Feline leukemia virus detection by immunohistochemistry and polymerase chain reaction in formalin-fixed, paraffin-embedded tumor tissue from cats with lymphosarcoma. Can. J. Vet. Res..

[6] McLuckie A.J., Barrs V.R., Lindsay S., Aghazadeh M., Sangster C., Beatty J.A. (2018). Molecular diagnosis of Felis catus Gammaherpesvirus 1 (FcaGHV1) infection in cats of known retrovirus status with and without lymphoma. Viruses.

[7] MacLachlan N., Dubovi E. (2017). Fenner’s Veterinary Virology.

[8] Sparkes A.H. (2003). Feline leukaemia virus and vaccination. J. Feline Med. Surg..

[9] Patel M., Carritt K., Lane J., Jayappa H., Stahl M., Bourgeois M. (2015). Comparative efficacy of feline leukemia virus (FeLV) inactivated whole-virus vaccine and canarypox virus-vectored vaccine during virulent FeLV challenge and immunosuppression. Clin. Vaccine Immunol..

[10] Jirjis F.F., Davis T., Lane J., Carritt K., Sweeney D., Williams J., Wasmoen T. (2010). Protection against feline leukemia virus challenge for at least 2 years after vaccination with an inactivated feline leukemia virus vaccine. Vet. Ther..

[11] Torres A.N., O’Halloran K.P., Larson L.J., Schultz R.D., Hoover E.A. (2010). Feline leukemia virus immunity induced by whole inactivated virus vaccination. Vet. Immunol. Immunopathol..

[12] Hofmann-Lehmann R., Cattori V., Tandon R., Boretti F.S., Meli M.L., Riond B., Pepin A.C., Willi B., Ossent P., Lutz H. (2007). Vaccination against the feline leukaemia virus: Outcome and response categories and long-term follow-up. Vaccine.

[13] Yamamoto J.K., Pu R.Y., Sato E., Hohdatsu T. (2007). Feline immunodeficiency virus pathogenesis and development of a dual-subtype feline-immunodeficiency-virus vaccine. AIDS.

[14] Westman M.E., Malik R., Hall E., Norris J.M. (2016). The protective rate of the feline immunodeficiency virus vaccine: An Australian field study. Vaccine.

[15] Hartaningsih N., Dharma D.M.N., Soeharsono S., Wilcox G.E. (2001). The induction of a protective immunity against Jembrana disease in cattle by vaccination with inactivated tissue-derived virus antigens. Vet. Immunol. Immunopathol..

[16] Ditcham W.G., Lewis J.R., Dobson R.J., Hartaningsih N., Wilcox G.E., Desport M. (2009). Vaccination reduces the viral load and the risk of transmission of Jembrana disease virus in Bali cattle. Virology.

[17] Sahay B., Yamamoto J.K. (2018). Lessons learned in developing a commercial FIV vaccine: The immunity required for an effective HIV-1 vaccine. Viruses.

[18] Yamamoto J.K., Sanou M.P., Abbott J.R., Coleman J.K. (2010). Feline immunodeficiency virus model for designing HIV/AIDS vaccines. Curr. HIV Res..

[19] Willett B.J., Hosie M.J. (2013). Feline leukaemia virus: Half a century since its discovery. Vet. J..

[20] Hartmann K. (2012). Clinical aspects of feline retroviruses: A review. Viruses.

[21] Westman M., Norris J., Malik R., Hofmann-Lehmann R., Harvey A., McLuckie A., Perkins M., Schofield D., Marcus A., McDonald M. (2019). The diagnosis of feline leukaemia virus (FeLV) infection in owned and group-housed rescue cats in Australia. Viruses.

[22] Hartmann K., Hofmann-Lehmann R. (2020). What’s new in feline leukemia virus infection. Vet. Clin. Small Anim..

[23] Flynn J.N., Dunham S.P., Watson V., Jarrett O. (2002). Longitudinal analysis of feline leukemia virus-specific cytotoxic T lymphocytes: Correlation with recovery from infection. J. Virol..

[24] Flynn J.N., Hanlon L., Jarrett O. (2000). Feline leukaemia virus: Protective immunity is mediated by virus-specific cytotoxic T lymphocytes. Immunology.

[25] Hoover E.A., Mullins J.I. (1991). Feline leukemia virus infection and diseases. J. Am. Vet. Med. Assoc..

[26] Hoover E.A., Schaller J.P., Mathes L.E., Olsen R.G. (1977). Passive immunity to feline leukemia: Evaluation of immunity from dams naturally infected and experimentally vaccinated. Infect. Immun..

[27] Torres A.N., Mathiason C.K., Hoover E.A. (2005). Re-examination of feline leukemia virus: Host relationships using real-time PCR. Virology.

[28] Hofmann-Lehmann R., Tandon R., Boretti F.S., Meli M.L., Willi B., Cattori V., Gomes-Keller M.A., Ossent P., Golder M.C., Flynn J.N. (2006). Reassessment of feline leukaemia virus (FeLV) vaccines with novel sensitive molecular assays. Vaccine.

[29] Jarrett O., Ganiere J.P. (1996). Comparative studies of the efficacy of a recombinant feline leukaemia virus vaccine. Vet. Rec..

[30] Hoover E.A., Mullins J.I., Chu H.J., Wasmoen T.L. (1996). Efficacy of an inactivated feline leukemia virus vaccine. AIDS Res. Hum. Retroviruses.

[31] Hofmann-Lehmann R., Holznagel E., Aubert A., Ossent P., Reinacher M., Lutz H. (1995). Recombinant FeLV vaccine: Long-term protection and effect on course and outcome of FIV infection. Vet. Immunol. Immunopathol..

[32] Lutz H., Addie D., Belák S., Boucraut-Baralon C., Egberink H., Frymus T., Gruffydd-Jones T., Hartmann K., Hosie M.J., Lloret A. (2009). Feline leukaemia: ABCD guidelines on prevention and management. J. Feline Med. Surg..

[33] Scherk M.A., Ford R.B., Gaskell R.M., Hartmann K., Hurley K.F., Lappin M.R., Levy J.K., Little S.E., Nordone S.K., Sparkes A.H. (2013). 2013 AAFP feline vaccination advisory panel report. J. Feline Med. Surg..

[34] Harbour D.A., Gunn-Moore D.A., Gruffydd-Jones T.J., Caney S.M.A., Bradshaw J., Jarrett O., Wiseman A. (2002). Protection against oronasal challenge with virulent feline leukaemia virus lasts for at least 12 months following a primary course of immunisation with Leukocell (TM) 2 vaccine. Vaccine.

[35] Westman M.E., Malik R., Hall E., Sheehy P.A., Norris J.M. (2017). Comparison of three feline leukaemia virus (FeLV) point-of-care antigen test kits using blood and saliva. Comp. Immun. Microbiol. Infect. Dis..

[36] Tandon R., Cattori V., Gomes-Keller M.A., Meli M.L., Golder M.C., Lutz H., Hofmann-Lehmann R. (2005). Quantitation of feline leukaemia virus viral and proviral loads by TaqMan (R) real-time polymerase chain reaction. J. Virol. Methods.

[37] Boenzli E., Hadorn M., Hartnack S., Huder J., Hofmann-Lehmann R., Lutz H. (2014). Detection of antibodies to the feline leukemia virus (FeLV) transmembrane protein p15E: An alternative approach for serological FeLV detection based on antibodies to p15E. J. Clin. Microbiol..

[38] Parr Y.A., Beall M.J., Leutenegger C., Levy J.K., Willett B.J., Hosie M.J. (2016). Investigating the Biology Underlying Discordancy in FeLV Diagnostics.

[39] Westman M.E., Malik R., Hall E., Sheehy P.A., Norris J.M. (2015). Determining the feline immunodeficiency virus (FIV) status of FIV-vaccinated cats using point-of-care antibody kits. Comp. Immun. Microbiol. Infect. Dis..

[40] Crawford P.C., Levy J.K. (2007). New challenges for the diagnosis of feline immunodeficiency virus infection. Vet. Clin. N. Am. Small Anim. Pract..

[41] Sebring R.W., Chu H.J., Chavez L.G., Sandblom D.S., Hustead D.R., Dale B., Wolf D., Acree W.M. (1991). Feline leukemia virus vaccine development. J. Am. Vet. Med. Assoc..

[42] Lehmann R., Franchini M., Aubert A., Wolfensberger C., Cronier J., Lutz H. (1991). Vaccination of cats experimentally infected with feline immunodeficiency virus, using a recombinant feline leukemia virus vaccine. J. Am. Vet. Med. Assoc..

[43] Legendre A.M., Hawks D.M., Sebring R., Rohrbach B., Chavez L., Chu H.J., Acree W.M. (1991). Comparison of the efficacy of three commercial feline leukemia virus vaccines in a natural challenge exposure. J. Am. Vet. Med. Assoc..

[44] Hofmann-Lehmann R., Hartmann K. (2020). Feline leukaemia virus infection: A practical approach to diagnosis. J. Feline Med. Surg..

[45] Lutz H., Pedersen N., Higgins J., Hubscher U., Troy F.A., Theilen G.H. (1980). Humoral immune reactivity to feline leukemia virus and associated antigens in cats naturally infected with feline leukemia virus. Cancer Res..

[46] Little S., Levy J., Hartmann K., Hofmann-Lehmann R., Hosie M., Olah G., Denis K.S. (2020). 2020 AAFP Feline Retrovirus Testing and Management Guidelines. J. Feline Med. Surg..

[47] Brunner C., Kanellos T., Meli M.L., Sutton D.J., Gisler R., Gomes-Keller M.A., Hofmann-Lehmann R., Lutz H. (2006). Antibody induction after combined application of an adjuvanted recombinant FeLV vaccine and a multivalent modified live virus vaccine with a chlamydial component. Vaccine.

[48] Shaw F.E., Guess H.A., Roets J.M., Mohr F.E., Coleman P.J., Mandel E.J., Roehm R.R., Talley W.S., Hadler S.C. (1989). Effect of anatomic injection site, age and smoking on the immune response to hepatitis B vaccination. Vaccine.

[49] Jin H., Xu Y., Shi F., Hu S. (2019). Vaccination at different anatomic sites induces different levels of the immune responses. Res. Vet. Sci..

[50] Jin H., Wu Y., Bi S., Xu Y., Shi F., Li X., Ma X., Hu S. (2020). Higher immune response induced by vaccination in Houhai acupoint relates to the lymphatic drainage of the injection site. Res. Vet. Sci..

[51] Hartmann K., Day M.J., Thiry E., Lloret A., Frymus T., Addie D., Boucraut-Baralon C., Egberink H., Gruffydd-Jones T., Horzinek M.C. (2015). Feline injection site sarcoma: ABCD guidelines on prevention and management. J. Feline Med. Surg..

[52] Hendricks C.G., Levy J.K., Tucker S.J., Olmstead S.M., Crawford P.C., Dubovi E.J., Hanlon C.A. (2014). Tail vaccination in cats: A pilot study. J. Feline Med. Surg..

[53] Grosenbaugh D.A., Leard T., Pardo M.C. (2006). Protection from challenge following administration of a canarypox virus–vectored recombinant feline leukemia virus vaccine in cats previously vaccinated with a killed virus vaccine. J. Am. Vet. Med. Assoc..

[54] Grosenbaugh D.A., Leard T., Pardo M.C., Motes-Kreimeyer L., Royston M. (2004). Comparison of the safety and efficacy of a recombinant feline leukemia virus (FeLV) vaccine delivered transdermally and an inactivated FeLV vaccine delivered subcutaneously. Vet. Ther..

[55] Schwartzkoff C.L., Egerton J.R., Stewart D.J., Lehrbach P.R., Elleman T.C., Hoyne P.A. (1993). The effects of antigenic competition on the efficacy of multivalent footrot vaccines. Aust. Vet. J..

[56] Offit P.A., Quarles J., Gerber M.A., Hackett C.J., Marcuse E.K., Kollman T.R., Gellin B.G., Landry S. (2002). Addressing parents’ concerns: Do multiple vaccines overwhelm or weaken the infant’s immune system?. Pediatrics.

[57] Wilson S., Saunders G., Stoeva M., Ludlow D., Von Reitzenstein M., Sture G., Salt J., Thompson J. (2014). Co-administration of an adjuvanted FeLV vaccine together with a multivalent feline vaccine to cats is protective against virulent challenge with feline leukaemia virus, calicivirus, herpes virus and panleukopenia virus. Trials Vaccinol..

[58] Poulet H., Brunet S., Boularand C., Guiot A.L., Leroy V., Tartaglia J., Minke J., Audonnet J.C., Desmettre P. (2003). Efficacy of a canarypox virus-vectored vaccine against feline leukaemia. Vet. Rec..

[59] Stickney A., Ghosh S., Cave N., Dunowska M. (2020). Lack of protection against feline immunodeficiency virus infection among domestic cats in New Zealand vaccinated with the Fel-O-Vax^®^ FIV vaccine. Vet. Microbiol..

